# Reviews

**Published:** 1887-01

**Authors:** 


### REVIEWS.

El colera en Valencia en 1885. Memoria acerca de los trabajos realizados durante la epidemia. * * * * presentada en nombre de La Junta Municipal de Sanidad. 8° Valencia. Imprenta de Manuel Alufre. 1886.

[Cholera in Valencia in 1885. A report upon the epidemic, etc., presented in the name of the Municipal Board of Health.]

The report before us is bound with paper, and besides 180 royal octavo pages, contains an appendix with numerous tabulated statements showing the course of the disease in its various aspects. At the end there are also plans of a cholera hospital erected in the suburbs in 1885, a good map of the city upon which the locations of the deaths from cholera are indicated, and an excellent chart by which the movement of the epidemic, both as to attacks and deaths, is graphically compared with the variations of the atmosphere, including changes in pressure, temperature, humidity, rainfall, etc.

The report, exclusive of the statistical portion, is divided into three parts, viz: clinical, hygienic, and administrative. In the clinical portion such subjects, as topography and geology, clinical history of the attacks, development of the epidemic and anticholeric inoculations, are discussed more or less fully.

The report states that "The facts observed in Valencia demonstrate in a very evident manner the non-transmissibility of cholera by air, except possibly at extremely short distances and in the midst of a very intense focus of infection.

"Transmission within the foci appears to be effected rather by contaminated clothing than by other means. In the first 150 cases, the foci were formed by importation, and in none of these first cases did the disease spread to the neighboring houses or even to the other rooms of the same house. Whenever the disease leaped to another house, it could always be proven that the person attacked therein had been in direct contact with the sick or had removed contaminated clothing to places in which the disease developed."

The reporters are of the opinion that the epidemic in Valencia also showed water to be a medium for spreading cholera, especially the water of sluggish and narrow streams:

"The irrigation canals performed the office of carrier (of the contagium of cholera), and in the interior of the city the sewers with a slow current contributed to the transmission and spread of the epidemic."

The reporters appear to accepted the doctrines of Koch concerning the etiology of cholera, but at the same time they collected some facts which cause them to admit also, with Pettenkofer, that there is a close relation between the level of the surface water and the outbreak of an epidemic.

They caused chemical and bacteriological examinations to be frequently made of the public water supply and of the water in private wells. Only in the latter was the presence of the comma bacillus recognized.

Altogether, this report is a credit not only to the members of the Board of Health of Valencia, but also to the municipality and to the printer, for it indicates a knowledge of modern medicine quite up with the times, it reflects the energy and public spirit which have made its publication possible, and superior typography leaves nothing to be desired. Moreover, it constitutes a valuable contribution to the history of the last epidemic of cholera in Europe. E. O. S.

Vade Mecum of Equine Anatomy, by A. Liautard. Second edition revised, New York, 1886. Published by Wm. R. Jenkins.

This useful little volume appears in better type and paper, a great convenience for rapid reading. With revision of the first edition, professor Liautard has added a table of muscular relations. It serves its place well, for rapid review of the student's studies, or for refreshing the memory in a moment of need. H.

The Equine Hospital Prescriber, by James B. and Albert Gresswell, London, 1886. Baillière, Tindall & Cox.

If this almanac collection of receipts have, as the authors claim, nearly all "proved to be of great value," we sincerely hope that those who are unable to formulate their own mode of treatment may derive great success in the treatment of "aneurysm, anguia pectoris, bots, diabetes mellitus, farcy, lampas, stringhalt," etc. We doubt if the book is of much value to others. H.

